# Telemedicine and reduction of travel-related environmental impact of digestive clinic care in a Canadian province

**DOI:** 10.1093/jcag/gwaf032

**Published:** 2025-12-17

**Authors:** Jared Morris, Desmond Leddin, Geoffrey C Nguyen, Harminder Singh, Charles N Bernstein

**Affiliations:** Department of Internal Medicine, Max Rady College of Medicine, Rady Faculty of Health Sciences, University of Manitoba, Winnipeg, Manitoba, R3A1R9, Canada; University of Manitoba IBD Clinical and Research Centre, Winnipeg, Manitoba, R3E3P4, Canada; Department of Medicine, Dalhousie University, Halifax, Nova Scotia, B3H4R2, Canada; Division of Gastroenterology and Hepatology, University of Toronto, Toronto, Ontario, M5S3H2, Canada; Department of Internal Medicine, Max Rady College of Medicine, Rady Faculty of Health Sciences, University of Manitoba, Winnipeg, Manitoba, R3A1R9, Canada; University of Manitoba IBD Clinical and Research Centre, Winnipeg, Manitoba, R3E3P4, Canada; Department of Internal Medicine, Max Rady College of Medicine, Rady Faculty of Health Sciences, University of Manitoba, Winnipeg, Manitoba, R3A1R9, Canada; University of Manitoba IBD Clinical and Research Centre, Winnipeg, Manitoba, R3E3P4, Canada

**Keywords:** telemedicine, convenience, carbon emission savings, sustainability

## Abstract

**Background:**

Telemedicine offers a promising approach to reduce the carbon footprint of healthcare delivery by minimizing travel-related greenhouse gas emissions. In this study, we quantified the carbon emissions savings from shifting gastroenterology clinic visits from in-person to telemedicine in a single gastroenterologist’s clinic in a major urban Canadian centre that serves a mixed urban and rural Canadian population.

**Methods:**

A cross-sectional analysis was conducted on 5690 telemedicine encounters from March 2020 to March 2022 at a tertiary-care gastroenterology clinic in Winnipeg, Manitoba, for a single gastroenterologist. Carbon emissions related to travel from home to clinic were estimated. The values are presented as CO2e, a standardized measure used to compare and aggregate the impact of different greenhouse gases on global warming. Travel distances were estimated using driving routes or flights for non-drivable locations. Clinic operational emissions were also estimated to assess total potential savings.

**Results:**

The total potential travel distance avoided was 880 336 km. Rural patients accounted for 92.7% of this distance. The average CO2e emissions saved per encounter was 42.9 kg, with rural encounters averaging 106.7 kg and urban encounters 4.6 kg. Clinic operational emissions were minimal at 0.06 kg of CO2e per encounter, compared to travel-related emissions. Over the 2 years, telemedicine visits saved approximately 244 079 kg of CO2e, underscoring the significant environmental benefit of virtual care.

**Conclusion:**

Telemedicine reduces the carbon footprint of gastroenterology outpatient care by minimizing patient travel, especially for rural populations. Incorporating telemedicine into routine practice can promote environmental sustainability within healthcare systems.

## Introduction

Global climate change is one of the most urgent challenges facing humanity, with adverse health, economic, and environmental effects.^1^ Healthcare delivery contributes substantially to greenhouse gas (GHG) emissions. Patient travel is a significant driver of the healthcare carbon footprint.^2^ Telemedicine can deliver quality care while reducing travel. Rapid adoption occurred during the COVID-19 pandemic due to social distancing and infection control.^3^

Telemedicine offers additional benefits. It can reduce waiting time for in-person visits, allow scheduling flexibility, and permit patients to remain at home or work with less disruption. It can lower out-of-pocket costs for fuel, parking, and lost work time. Environmental gains include reductions in carbon dioxide and other emissions.^4^ Reducing GHGs is central to climate mitigation and public health. Cleaner energy sources also improve health outcomes.^5^

Digestive health care often requires repeat clinic visits for chronic disease management, review of endoscopy and pathology, and patient education. These visits can impose substantial travel burdens, especially for rural patients who travel long distances to urban specialty clinics.^6^^,^^7^ Data on travel-related emissions for gastroenterology follow-up are limited. This study quantifies emissions associated with travel to gastroenterology clinics and estimates the avoided emissions when visits occur by telemedicine and when the clinic space is not used for in-person care.

The aims of this study were to quantify the reductions in carbon emissions resulting from travel avoided to and from a tertiary-care gastroenterology clinic, and to estimate the additional carbon savings attributable to reduced outpatient clinic operations during the study period.

## Methods

### Study design and setting

This was a cross-sectional analysis of appointments at a single gastroenterology clinic run by 1 practitioner at a tertiary-care hospital in Winnipeg, Canada. The study period was March 2, 2020, to March 28, 2022, a time when telemedicine was heavily used. During this period, nearly all in-person physician visits were replaced by telephone encounters. Encounters with clerical or nursing staff were not counted.

### Study population

All patients with at least 1 telemedicine encounter during the study period were eligible. Encounters were identified from clinic sheets for the physician. Each eligible encounter was treated as 1 data point for emission calculations.

### Inclusion criteria

Adult patients (≥18 years of age) who completed at least 1 telemedicine encounter during the study period at the gastroenterology clinic with the clinician were included.

### Exclusion criteria

Patients were excluded if they did not have a fixed home address (preventing distance calculation from home to clinic), if they were unable to complete a virtual assessment or required an in-person visit for clinical reasons.

Patients were excluded if there were unable to compete a virtual assessment or had a clinical need for an in-person visit or resided outside of Manitoba.

Patients were excluded if there were unable to compete a virtual assessment or had a clinical need for an in-person visit or resided outside of Manitoba.

### Classification of urban versus rural

Patients with home addresses within Winnipeg city limits were classified as urban. Those outside city limits were classified as rural. Locations were verified using publicly available mapping tools.

### Distance collection

The clinic provides specialist care to patients all over Manitoba, and others from Northern Ontario and Nunavut. Manitoba is over 500 000 km^2^ in area.^8^ An assumption was made that every patient with a drivable road to Winnipeg drove. There is no commuter train service for the Winnipeg area. Patients with no drivable road were assumed to have flown. This does not significantly affect the overall data due to the relatively close values of average car emissions to average flight emissions for a domestic flight.^9^ As this is a model, it is assumed the flights are equivalent makes and models to the flights used in the UK database. Distance to the clinic was determined based on the driving route from home addresses using Google maps. Flight distance was found using a distance calculator for flights^10^ to calculate the flight distance between airports.

### Carbon emission calculations

The distance from home to clinic needed to be converted to carbon emissions. The values are presented as CO2e, which is a standardized measure used to compare and aggregate the impact of different GHGs on global warming.^1^ To calculate the average emission factor for urban and rural encounters, a random sample of 100 patient encounters from each group was telephoned to identify the make, model, and year of the vehicles likely used. These vehicles were then categorized into small, medium, and large sizes, each associated with emissions per kilometer (kg of CO2e/km) based on UK government data.^9^ The proportion of vehicles in each size category was calculated and multiplied by their respective emissions per kilometer to yield a weighted average. These weighted averages were used as the adjusted conversion factors, representing the typical emissions per kilometer for each group.

For flights, the UK source^9^ was again used to quantify the kg of CO2e per km using domestic flight data, and used as a conversion factor. As per the previous assumption, all patients who had no drivable route as per Google Maps were assumed to have flown. With this, CO2e emissions in kg of CO2e could be calculated using the following equation:


Kg of CO2e per encounter=Distance one way x 2 x urban or rural or flight conversion factor

where “distance one way” is the driving distance from home address to clinic, and “2” accounts for the return trip. The urban or rural conversion factor represents the weighted average of the CO2e values from the respective sample vehicles described above. The flight conversion factor is the value listed in the UK source for domestic flights. The sum of these encounter values represents the CO2e emissions for all telemedicine encounters.

### Public transit

Since an assumption was made that every patient with a drivable road drove to their appointment, a second model was developed to show the emissions if public transit was used as well. In 2022, 6% of Manitoba’s population used public transit to commute^11^. Most rural Manitoba towns do not have a commuter train or bus service into the city of Winnipeg; therefore, public transit emissions were only calculated within the urban setting. Since there is no commuter train service in Winnipeg, public transit emissions in an urban setting were represented as bus emissions, as they would be the sole contributor. The public transit emissions were modeled by taking 6% of the total urban distance and multiplying it by the kg of CO2e per km for bus emissions.

### Clinic operational emissions

Emissions associated with running an in-person clinic need to be considered. Staff engineers from the Province of Manitoba health governing body Shared Health provided values for running a 92.9 m^2^ (1000 ft^2^) ambulatory clinic over the study period. The clinic space used by the physician was 40.88 m^2^ (440 ft^2^), which accounted for 5 patient rooms, equipped with a desk, computer, and examination table. The physician potentially used the clinic for a maximum of 2 days per week, for 9 hours each day. Therefore, the emissions associated with the clinic will be reduced to account for being active 2 out of 7 days per week, for 9 out of 24 hours.

The emission data provided by Shared Health were broken down into base electric, cooling electric, base thermal, heating thermal, and total energy. The base values represented the emissions if the clinic was not being used. Cooling electric and heating thermal were the values added when the clinic was running and full commission.

### Ethics

Medical studies that exclusively rely on publicly available information and anonymous data, and are for quality assurance, program evaluation, or performance reviews, do not require ethics approval. This was discussed with and confirmed with the University of Manitoba Health Research Ethics Board.

## Results

### Study population

**Figure 1.**
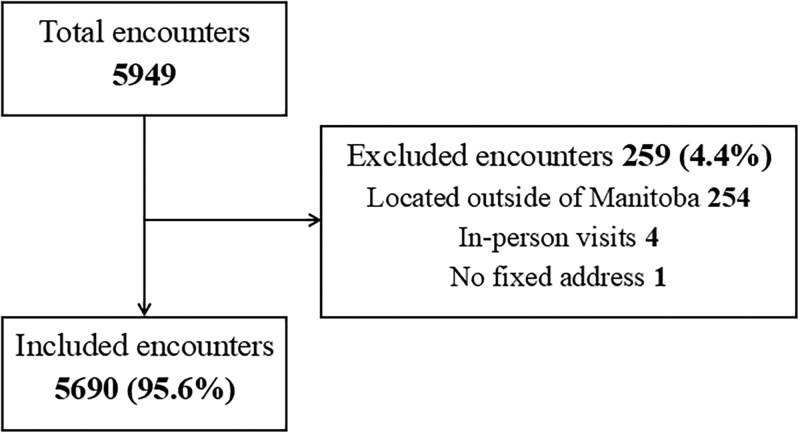
Flow diagram of telemedicine encounter inclusions.

There were 5949 telemedicine encounters among 2909 patients during the study period. After applying exclusion criteria, 5690 telemedicine encounters remained for analysis (Figure 1). Of these, 3559 encounters (62.6%) were urban and 2131 (37.4%) were rural (Table 1).

**Table 1.** Demographic distribution of telemedicine encounters by urban and rural subgroup.

Group	Number of encounters	**Male** ***n* (%)**	**Female** ***n* (%)**	Mean age (years)	Median age (years)
**Urban**	3559	1540 (43.3)	2019 (56.7)	49.9	49.0
**Rural**	2131	886 (41.6)	1245 (58.4)	50.9	50.0
**Total**	5690	2426 (42.6)	3264 (57.4)	50.3	49.0

### Distance traveled

The total potential round-trip distance avoided across 5690 encounters was 880 336 km. Urban encounters accounted for 64 036 km (7.3%), and rural encounters accounted for 816 300 km (92.7%). The mean round-trip distance per encounter was 154.7 km. The urban mean was 18 km, and the rural mean was 383.1 km (Table 2).

**Table 2.** Distances for urban and rural subgroups.

Group	Distance travelled in km (2-way trip)	Mean distance (km)
**Urban**	64 036.3	17.9
**Rural**	816 299.8	383.1
**Total**	880 336.1	154.7

### Travel emissions

The total potential emissions from these avoided round trips were 243 764 kg CO2e. Urban encounters accounted for 16 393 kg CO2e (6.7%), and rural encounters accounted for 227 371 kg CO2e (93.3%). Mean emissions saved per encounter were 42.84 kg CO2e overall, 4.6 kg CO2e for urban encounters, and 106.7 kg CO2e for rural encounters. Table 3 presents the emissions per kilometer and weighted averages. Conversion factors used for calculating emissions are provided in Table 4. The breakdown of vehicle sizes in relation to emissions per km is listed in Table 5. Table 6 demonstrates the emissions of air travel.

**Table 3.** Urban and rural weighted averages based on random sampling and car size.

Group	**Vehicle size** ^a^	**Emissions per kilometer (kgCO2e/km)** ^a^	Percentage of patients	Attributed emissions (kgCO2e/km)
**Urban**	Large	0.28295	0.75	0.2122125
	Medium	0.19228	0.13	0.0249964
	Small	0.15371	0.12	0.0184452
**Weighted average**				0.2556541
**Rural**	Large	0.28295	0.97	0.2744615
	Medium	0.19228	0.02	0.0038456
	Small	0.15371	0.01	0.0015371
**Weighted average**				0.2798442

a Emissions per kilometer were chosen based on the UK emissions data. Flight and bus emissions were also taken from the UK emissions data.^9^

**Table 4.** Conversion factors for vehicles, flights, and buses.

Emission type	**Conversion factors (kgCO2e/km)** ^a^
**Urban**	0.25565
**Rural**	0.27984
**Flights (domestic)**	0.12076
**Buses**	0.25493

a Emissions per kilometer were chosen based on the UK emissions data. Flight and bus emissions were also taken from the UK emissions data.^9^

**Table 5.** Emissions for urban and rural groups, alongside subgroup emissions of flights.

Group	Emissions (kgCO2e)	Mean emissions (kgCO2e)
**Urban**	16 393.29 (6.73%)	4.61
**Rural** ^a^	227 371.21 (93.27%)	106.70
**Total**	243 764.50	42.84

a Within the rural group, a subset of encounters involved air travel (flights), and within the urban group, a subset involved public transit. These are illustrated separately in this table.

**Table 6.** Subgroup emissions of buses and flights.

Subgroup	Emissions (kgCO2e)	Mean emissions (kgCO2e)
**Flights (subgroup of rural)**	23 131	336.96
**Buses (subgroup of urban)** ^a^	463.98	-

a Since buses were calculated using 6% of the urban distance, there is no value for mean emissions, since only one value was used.

**Table 7.** Emissions for the urban group if buses are used, compared to if buses are not used.

Group	Emissions (kgCO2e)	Mean emissions (kgCO2e)
**Urban (no buses)**	16 393.29	4.61
**Urban (buses)**	15 873.67	4.46

### Public transit

Bus emissions are 0.12076 kg of CO2e per km per passenger.^9^ Six percent of the urban distance is 3842 km, and converted to bus emissions this is 464 kg of CO2e (Table 6). Accounting for bus use of 6% the new total urban emissions would be 15 874 kg of CO2e, or 4.46 kg of CO2e per urban encounter as compared to 4.6 kg if bus travel is not factored in, a difference of 3%. This is illustrated in Tables 6 and 7.

### Clinic operational emissions

The total potential emissions saved by running an in-person 40.88 m^2^ (440 ft^2^) ambulatory clinic over the study period was 2939.2 kg of CO2e. After accounting for 2 clinic days per week, for 9 hours per day, the effective value is 314.91 kg of CO2e. This equates to an average of 0.06 kg of CO2e per encounter.

Operational clinic emission values are summarized in Table 8. The base electric and base thermal values are present regardless of whether the clinic is occupied or not. The summation of these values over the study period represents CO2e emissions when a clinic is left idle, which is 814 kg of CO2e. Cooling electric and heating thermal represents the CO2e emissions required for a fully functional ambulatory clinic but does not include the base values. The emissions for cooling electricity were estimated to be 0 by the Shared Health provider. This is because air-conditioning is an electrical load. Manitoba electricity is generated almost entirely from renewable hydroelectricity with virtually no emissions, if the energy source is from Manitoba Hydro electrical grid.^12^^,^^13^ The emissions for heating thermal were 2939.2 kg of CO2e emissions. These emissions are primarily from burning natural gas to heat and ventilate the building. The total potential emissions to have an in-person 40.9 m^2^ (440 ft^2^) ambulatory clinic is 3753.2 kg of CO2e. However, for the purposes of the study, only the carbon emissions potentially saved are required. Therefore, heating thermal best estimates the potential carbon emissions saved by conducting telemedicine visits, which was 2939 kg of CO2e over 2 years, reduced to 314.91 kg of CO2e after accounting for working hours. Breaking this down further, the average clinic emissions per encounter is 0.06 kg of CO2e.

**Table 8.** Greenhouse gas emissions per 40.88 m^2^ (440 ft^2^) from March 2020 to March 2022, representing clinic space used.

Emission type	Emission value (kgCO2e)	Variables included
**Base electric** ^a^	8.8	HVAC, Heat recovery chiller, Lighting, Equipment (medical, IT, office equipment)
**Cooling electric** ^a^	0	Ventilation systems, Cooling plant
**Base thermal** ^a^	805.2	Ventilation systems, Heating plant
**Heating thermal** ^a^	2939.2	Ventilation systems, Heating system & Building envelope
**Total** ^a^	3753.2	

a Manitoba Hydro GHG emission factors are not significant for University of Manitoba. Over 99% of the building related GHGs are from natural gas. Manitoba Hydro electricity is renewable with minimal greenhouse gas emissions.^12^^,^^13^

### Net carbon savings

The total potential CO2e saved by converting an in-person clinic to telemedicine was 244 079 kg over 2 years for 1 physician, which equals 42.9 kg CO2e per virtual visit when operational savings are included. Incorporating 6 percent public transit for urban trips reduces the total to 243 245 kg CO2e and 42.81 kg CO2e per encounter, a difference of 0.21%.

## Discussion

An average gastroenterology appointment saved 42.9 kg CO2e when conducted virtually rather than in person. The mean saving for urban encounters was modest at 4.6 kg CO2e per visit, while rural encounters saved 106.7 kg CO2e per visit. Rural patients represented 37% of encounters but accounted for more than 93% of travel-related emissions avoided. This reflects the long travel distances often required to access specialist care in centralized, urban settings.

Our estimate is higher than the 14.9 kg CO2 per encounter reported by Galts et al.^7^ Their study had a mean round-trip distance of 57.3 km, much shorter than our mean of 154.7 km. Population density offers context, as the population density in Manitoba, Canada, is 2.5 people per square kilometer.^8^ The study by Galts et al was done in Southern Ontario^7^ which has a population density of 15.9 people per square kilometer.^14^ In contrast, Netherlands has a population density of 518 people per square kilometer.^15^ Lower density increases average travel distance, which increases emissions. Some difference also relates to metric choice, as Galts et al reported CO2, not CO2e.^7^ CO2e includes other GHGs and therefore yields a higher value for the same trip.^1^

Our data reflect a clinic serving a mixed rural and urban population. Savings for a purely urban practice would be lower. Over 2 years, 3559 urban encounters produced 16 393 kg CO2e (6.7%), which equals 4.6 kg per visit with an average round trip of 18 km. This is a stark reduction from the average total emissions of 42.9 kg of CO2e. The Galts’ data are based out of Hamilton, Ontario,^7^ which is also an urban population, and they may serve less of a rural or distant population. Their data are more comparable to our urban data.

Our findings overall do align with earlier research, such as from Purohit et al, who systematically reviewed the carbon footprint of telemedicine and identified that telemedicine services could save between 0.7 and 372 kg of CO2e per consultation.^4^ The variability in all our carbon savings reflects the context-dependent nature of telemedicine, shaped by factors like geographical location, and type of consultation method used (eg, video versus telephone).^4^

Zurl et al^16^ assessed travel-related carbon emissions for healthcare on a national scale in the United States. Rural patients generate higher emissions per trip due to greater travel distances; however, urban patients generated most total emissions because more healthcare trips were made.^16^ This shines light into what the emission data on a national scale may look like for Canada. Their modeling also demonstrated that there are interventions beyond telemedicine that could reduce emissions. If 30% of patients used electric vehicles, emissions declined by 23%. If 50% adopted electric vehicles, emissions declined by 38%.^16^ Incorporating these insights strengthens the argument that telemedicine not only enhances access and convenience but is also instrumental in advancing the environmental sustainability of healthcare delivery across diverse populations and geographic settings.

The environmental impact of healthcare extends beyond patient travel. The healthcare sector is responsible for approximately 4.6% of annual GHG emissions in Canada.^17^ Facility energy use and the production and disposal of medical equipment and pharmaceuticals contribute substantially.^7^ Telemedicine reduces emissions from facility operations by lowering on-site patient care demands and from travel logistics by reducing trips, thereby decreasing the overall environmental footprint of healthcare. In addition to this, Welk et al^18^ investigated the cost-saving benefits of telemedicine through virtual care in Ontario, Canada. There were significant patient-level cost savings related to gasoline, parking, and public transit expenses. These were more favourably benefiting rural residents, higher comorbidity patients, and those above the age of 65, as they had the highest distance traveled and emissions saved.^18^ Their work supports maintaining a stable level of virtual care to realize ecological and economic gains.^18^

Clinical outcomes have been investigated in relation to telemedicine. A systematic review of randomized controlled trials exploring telephone compared with video visits found no differences for the management of patients with an established diagnosis. Further research is required to determine if there are outcome differences between phone and in-person visits.^19^ Gordon et al^20^ found that web-based telehealth for adults with inflammatory bowel disease was likely comparable to usual care for symptom control, relapse prevention, and quality of life, with improved medication adherence. When combined with the environmental benefits as we have shown, this emphasizes integrating telehealth into standard care practices, as healthcare systems can further reduce their environmental impact while simultaneously improving patient outcomes.^20^ In our study, only 4 patients had subsequent in-person encounters following telephone visits, and no further data were collected regarding the reasons for these visits, as they were excluded. Although adverse clinical outcomes related to telemedicine were not assessed, this represents an area for future investigation to ensure that virtual care does not result in delayed or repeat appointments that could offset its environmental benefits.

Operational clinic emissions were negligible relative to travel emissions. Avoided operational emissions during the study period totaled 315 kg CO2e over 2 years, which is less than 0.2% of total savings. This shows that clinic emissions pale in comparison to travel-related emissions. Thiel et al reported virtual visits generate <1% of the GHGs relative to in-person visits. Their range was 0.02-0.08 kg of CO2e for virtual visits, depending on the department (eg, ENT, surgery, orthopedics, etc).^21^ Our study estimated 0.06 kg of CO2e per encounter, representing in-person emissions. Our low clinic emissions reflect Manitoba hydroelectricity being a source of renewable energy, as such not contributing to our GHG emissions.^12^^,^^13^

One limitation of our study is the average carbon emissions for vehicles driven by rural and urban patients were determined by random sampling of patients. This approach could under or overestimate true emissions. None of the patients in the random sample reported using electric or hybrid vehicles, which may have also led to an overestimation of travel-related emissions. While this study reflects a single practice at a tertiary referral centre, it is similar to other gastroenterology practices in Manitoba where a blend of rural and urban patients is seen. A significant strength of our study is the inclusion of a modeled estimate of public transit emissions within the urban subgroup. Given that 6% of Manitoba’s urban population uses public transit for commuting,^11^ we accounted for this by applying bus emission factors to 6% of the urban travel distance. This enhances the ecological validity of our findings by acknowledging that not all urban patients would travel by private vehicle. Our results showed that factoring in public transit reduced urban emissions by only 3%, and a marginal overall impact on total emissions saved (0.21%).

Based on the above information, our data of average CO2e emissions saved can be used as a model for other healthcare centres and extrapolated for both urban and rural populations to investigate carbon emissions. This Manitoba-based study is supported by the findings of both a Hamilton, Ontario-based study alongside a southern Ontario study by Welk et al.

In conclusion, telemedicine can significantly reduce the carbon footprint associated with healthcare delivery by minimizing travel. As we continue to navigate the challenges posed by climate change, integrating telemedicine into standard practice not only improves healthcare access but also contributes to the environmental sustainability of healthcare systems.

## Data Availability

The datasets presented in this article are not readily available. All available data are presented herein.
